# NLRP3 inflammasome and hearing loss: from mechanisms to therapies

**DOI:** 10.1186/s12974-025-03561-w

**Published:** 2025-10-04

**Authors:** Silvia Murillo-Cuesta, Elena Seoane, Blanca Cervantes, Jose Manuel Zubeldia, Isabel Varela-Nieto

**Affiliations:** 1 Instituto de Investigaciones Biomédicas Sols-Morreale (CSIC-UAM), Madrid, España; 2 Centro de Investigación Biomédica en Red en Enfermedades Raras (CIBERER), Madrid, España; 3 Instituto de Investigación Sanitaria del Hospital La Paz (IdiPAZ), Madrid, España; 4 Allergy Service, Hospital General Universitario Gregorio Marañón, Madrid, Spain; 5 Gregorio Marañón Health Research Institute (IiSGM), Madrid, Spain; 6 Dept. of Medicine, Complutense University School of Medicine, Madrid, Spain; 7 Escuela de Medicina, Universidad Anáhuac Puebla, Puebla, México

**Keywords:** Autoinflammatory diseases, Cryopyrin-associated periodic syndromes, Deafness, IL-1 receptor, Pyroptosis, Rare diseases

## Abstract

The NLRP3 inflammasome, a key component of the innate immune system, has emerged as a central mediator of inflammation-driven sensorineural hearing loss (SNHL). This review synthesizes current evidence on its involvement across a wide spectrum of auditory pathologies, including genetic syndromes such as CAPS and autosomal dominant deafness (DFNA) 34, as well as noise-induced, age-related, drug-induced, and viral hearing loss. Dysregulated activation of NLRP3 in the cochlea leads to the release of proinflammatory cytokines (IL-1B and IL-18) and pyroptotic cell death, contributing to irreversible cochlear damage.

Experimental studies have demonstrated that pharmacological inhibition of NLRP3 via agents such as MCC950, oridonin and tranylcypromine can preserve auditory function. Gain-of-function mutations in the NLRP3 gene are frequently associated with both syndromic and nonsyndromic hearing loss, and animal models expressing these mutations replicate cochlear inflammation and hearing deficits, validating their pathogenic role. Clinically, IL-1B signaling blockers such as anakinra and canakinumab have shown efficacy in CAPS patients, stabilizing or improving hearing outcomes. Emerging selective NLRP3 inhibitors, including dapansutrilo and MCC950, are progressing through early-phase clinical trials. Additionally, natural compounds such as piceatannol and oridonin have demonstrated otoprotective effects in preclinical models of noise- and drug-induced hearing loss.

Collectively, these findings position the NLRP3 inflammasome as a promising therapeutic target for SNHL. Future translational research should focus on validating NLRP3-targeting compounds in human trials, identifying biomarkers for early diagnosis, and exploring combination therapies that integrate anti-inflammatory, antioxidant, and regenerative strategies. Targeting NLRP3 may ultimately redefine treatment paradigms for preventing or halting progressive hearing loss.

**Graphical Abstract:**

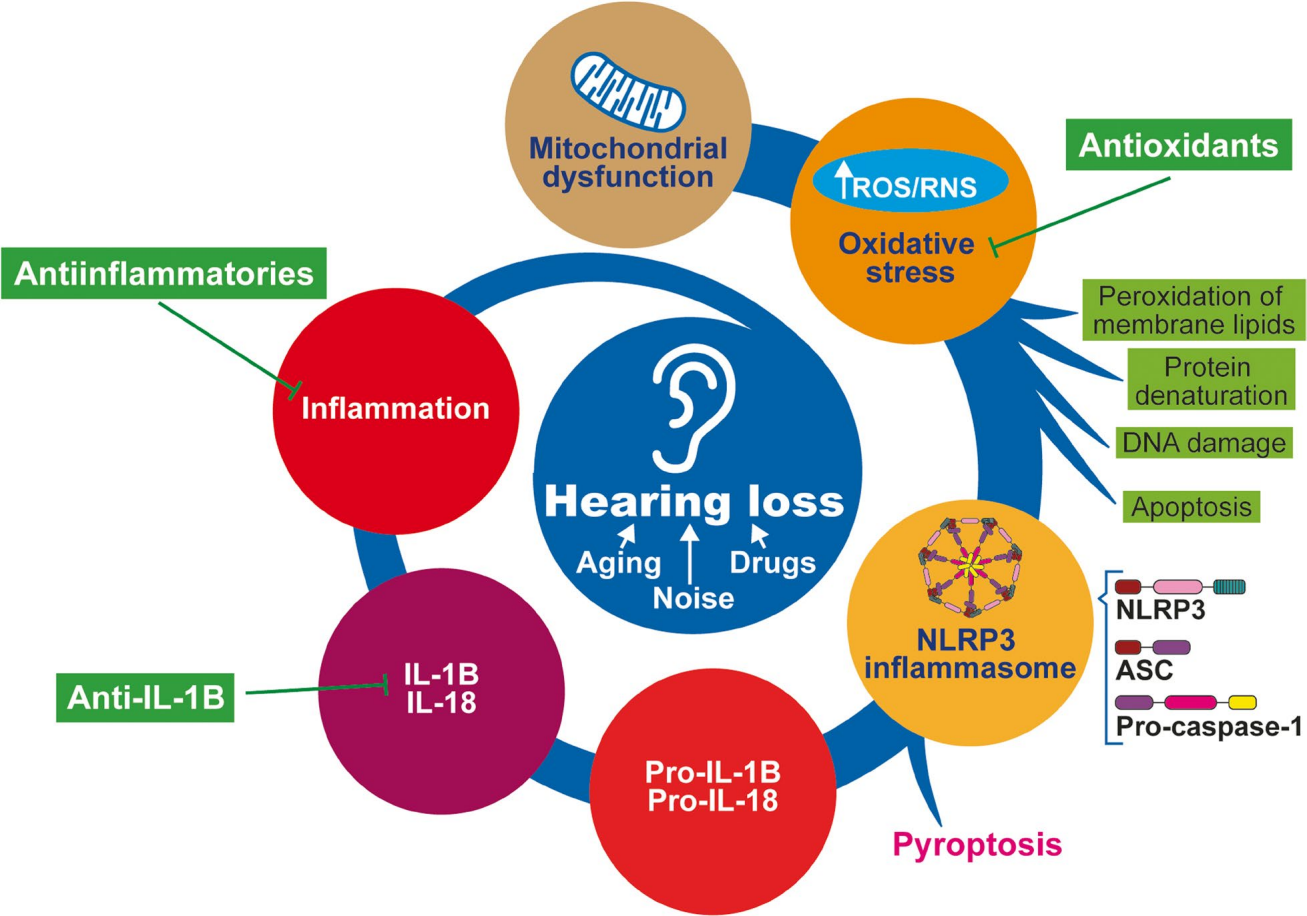

## Background

Inflammasomes are multiprotein complexes that assemble and activate in the cell cytoplasm in response to a variety of noxious signals, leading to cytokine release, which is the first step of the host defense response. The dysregulation of inflammasomes, mostly NOD-, LRR- and pyrin domain-containing protein 3 (NLRP3), has been traditionally associated with autoinflammatory syndromes, but in recent years, many studies have associated this structure with many other human diseases, including cardiovascular (stroke and atherosclerosis), allergic (asthma and urticaria) and neurodegenerative (Parkinson and Alzheimer) diseases, diabetes and cancer [1, 2]. Therefore, NLRP3 has emerged as a potential target for therapeutic intervention. The only FDA-approved medications for treating NLRP3-related diseases are inhibitors of interleukin-1beta (IL-1B) signaling, such as IL-1B (canakinumab and rilonacept) and IL-1R1 (anakinra) blockers (Drugs@FDA, accessed July 2025). Several other NLRP3 inflammasome inhibition strategies are currently under investigation, some of which have already entered clinical trials [3].

The NLRP3 inflammasome also seems to play an important role in the pathogenesis of different types of hearing loss, including chronic otitis media, rare genetic syndromic and nonsyndromic deafness, noise- or ototoxic-induced hearing impairment, presbycusis and tumor-induced hearing loss in vestibular schwannoma [4]. However, the precise mechanisms of NLRP3 inflammasome activation and their effects have not yet been clearly determined.

Here, we review the relationship between the NLRP3 inflammasome and hearing loss, summarize some of the relevant experimental results reported in recent years, and introduce promising NLRP3 inhibitors with potential use for clinical treatment.

## NLRP3 inflammasome and the cochlea

The NLRP3 inflammasome is a cytosolic protein complex that acts as an intracellular innate immune sensor and is composed of three basic elements: a pattern recognition receptor (NLRP3), an adaptor protein (apoptosis-associated speck-like protein containing a CARD, ASC), and an effector protease (caspase-1) [3].

**Fig. 1:**
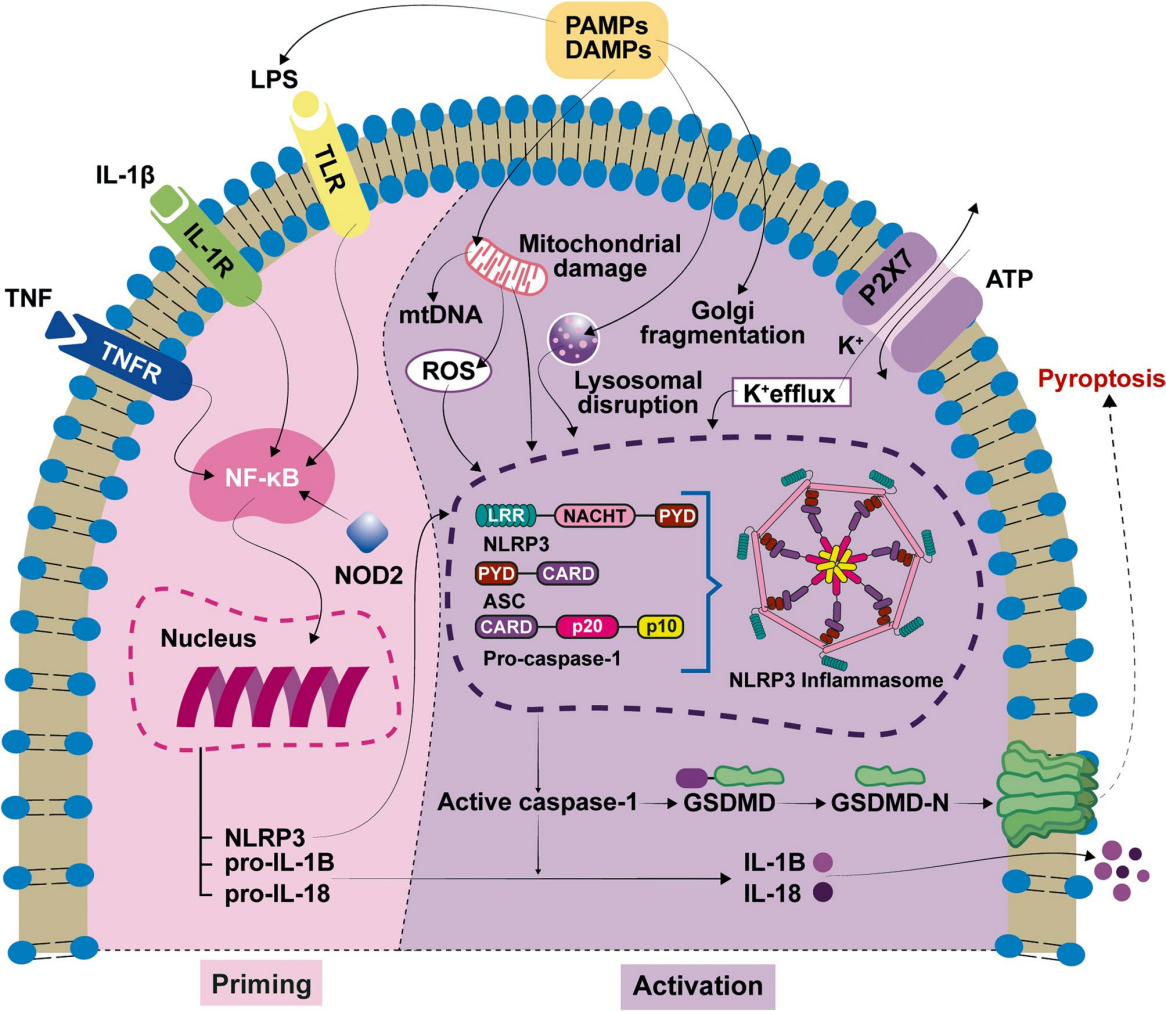
Although NLRP3 is expressed mainly in immune system cells (macrophages, neutrophils and lymphocytes), other cell types, including neurons, microglia, osteoblasts, and dendritic or epithelial cells, are also able to produce NLRP3 inflammasome components. Most of these cells are present in the mouse cochlea; therefore, *Nlrp3*, *Pycard* (encoding ASC), *Casp1* and *Il1b* mRNAs have been detected via RT‒qPCR [5]. NLRP3 is present mostly in resident macrophages at the stria vascularis and basilar membrane and is activated by classical stimuli, such as lipopolysaccharide (LPS) and ATP, leading to IL-1B secretion [5]. Single-cell RNA-seq studies of human and mouse inner ears show that *Nlrp3* is not highly expressed in sensory hair cells [6, 7]. Other authors have detected NLRP3 immunostaining in the spiral ganglion neurons of the mouse cochlea [8], which could be responsible for the spiral ganglion neuron death caused by pyroptosis observed in cytomegalovirus-induced hearing loss [9]. *Nlrp3* expression has also been confirmed in supporting cells by RNA-seq studies and validated by immunofluorescence [10]. Finally, increased expression of NLRP3, caspase-1, IL-1B, and gasdermin D (GSDMD) has been confirmed in the marginal cells of the stria vascularis in vitro [11]. In summary, NLRP3, together with other inflammasome components, is expressed in different cochlear cell types and constitutes a potential initiator of inflammatory responses in the hearing organ

NLRP3 inflammasome formation occurs in two steps: priming and activation. During priming, the detection of pathogen- and damage-associated molecular patterns (PAMPs and DAMPs) by different receptors (TLR, NOD2, IL1R1 or TNFR) results in the transcriptional upregulation of NLRP3 and proinflammatory cytokines. Inactivated NLRP3 remains as a monomer or oligomer and localizes to membranes. The activation stage occurs when NLRP3 recognizes a secondary damaging stimulus and then recruits ASC monomers, forming a filamentous structure (called a speck), upon which caspase-1 is activated. Activated caspase 1 processes the procytokines IL-1B and IL-18 into their proinflammatory forms and gasdermin D, which induces pore formation in the plasma membrane and ultimately cell death via pyroptosis. IL-1B and IL-18 exit the cell through these pores and bind to their receptors (IL-1R1 and IL-18-R1/IL-18-RAP, respectively) on surrounding cells, eliciting downstream inflammatory responses, including the production of other inflammatory mediators, such as IL-6 (Fig. 1).

The activation of the NLRP3 inflammasome follows a two-signal model. First, a priming signal (PAMPs like LPS or DAMPs such as TNF-α) activates pattern recognition receptors (like TLRs), leading to NF-κB activation. This transcription factor upregulates the expression of pro-IL-1B, pro-IL-18, and NLRP3. Next, a second activation signal (potassium efflux, reactive oxygen species, mitochondrial damage, or lysosomal rupture) induce NLRP3 oligomerization, allowing it to recruit the adaptor protein ASC, which in turn binds pro-caspase-1. The assembled inflammasome catalyzes the cleavage of pro-caspase-1 into its active form, enabling the processing of pro-cytokines into their mature form, and the cleavage of GSDMD, whose N-terminal fragment forms pores in the cell membrane, leading to pyroptotic cell death. Abbreviations: ASC, apoptosis-associated speck-like protein containing a CARD; CARD, caspase activation and recruitment domain; DAMPs, damage-associated molecular patterns; GSDMD, gasdermin D; GSDMD-N, gasdermin D N-terminal; IL-1B, interleukin-1B; IL-18, interleukin-18; mtDNA, mitochondrial DNA; NACHT, an acronym from NAIP, CIITA, HET-E and TP1; NF- κB, nuclear factor kappa-light-chain-enhancer of activated B cells; NLRP3, NLR family pyrin domain-containing 3; NOD-2, nucleotide-binding oligomerization domain-containing protein 2; LPS, lipopolysaccharide; LRR, leucine-rich repeat; PAMPs, pathogen-associated molecular patterns; PYD, pyrin domain; ROS, reactive oxygen species; TNF, tumor necrosis factor; TNFR, tumor necrosis factor receptor; TLR, toll-like receptor.

## NLRP3 and hearing loss

Sensorineural hearing loss (SNHL) affects approximately 1.5 billion people globally. Approximately 430 million of these patients require rehabilitation services for disabling hearing loss [12]. SNHL is caused mainly by dysfunction of the inner ear due to aging, exposure to ototoxic drugs or noise, or mutations in nuclear or mitochondrial genes. However, it is idiopathic in some patients. Although the inner ear was previously thought of as an immune-privileged organ, inflammation—along with oxidative stress—is considered a central pathogenic mechanism of hearing loss [13, 14].

The activation of the NLRP3 inflammasome constitutes a physiological host defense response to danger signals. However, dysregulated inflammasome activity can lead to excessive inflammation, causing substantial damage, especially in those tissues, such as the cochlea, with scarce regenerative capacity. There is increasing evidence that inflammasome activation is associated with hearing loss. Thus, gain-of-function *NLRP3* mutations in autoinflammatory diseases are commonly associated with hearing loss. Furthermore, the increase in the cochlear uptake of gadolinium, measured as an increased intensity of the MRI signal, indicates barrier leakage and is normalized by anakinra therapy. The induction of the NLRP3 inflammasome has also been confirmed in experimental models of noise-induced hearing loss [15], ototoxicity induced by aminoglycoside antibiotics [16] or platin-derived chemotherapeutics [11]. In addition, hearing loss can occur due to cytomegalovirus infection during pregnancy [17].

### Hearing loss in cryopyrin-associated periodic syndrome

Abnormal hyperactivation of the inflammasome and excessive production of IL-1B are the causes of a spectrum of autosomal dominant systemic autoinflammatory diseases called cryopyrin-associated periodic syndrome (CAPS), which include (in order of severity) familial cold autoinflammatory syndrome (FCAS), Muckle–Wells syndrome (MWS), and chronic infantile neurological, cutaneous and articular (CINCA) syndrome. These diseases have an estimated world prevalence of 2.7–5.5 per 1 million people [18] and are considered ultrarare diseases (ORPHA:208650). However, considering that many patients are diagnosed very late or not at all, its prevalence is likely greater.

The typical inflammatory symptoms observed in CAPS include fever, headache or fatigue, and local symptoms in the skin, joints, muscles, eyes, cochlea and central nervous system [19]. The correct diagnosis and immediate initiation of therapy with IL-1B inhibitors are mandatory in most patients to achieve a reversal of daily symptoms and prevent possible life-threatening sequelae [18].

FCAS was first described in 1940 in a five-generation family exhibiting recurrent episodes of urticarial-like rash, limb pain and fever following cold exposure [20]. Over the next few decades, similar cases with “cold hypersensitivity” were reported and finally classified as FCAS to differentiate this inherited disorder from the more common acquired cold urticaria. In addition to urticaria rash and a burning sensation triggered by exposure to cold, fever, malaise, conjunctivitis, abdominal discomfort and polyarthralgias are very common, whereas amyloidosis and deafness are uncommon or absent.

MWS presents similar symptoms (fever, rash, arthralgia, conjunctivitis, amyloidosis) and sensorineural deafness. The first report of the auditory phenotype in MWS was in 2012, when a single-center MWS cohort (19 patients aged 3–72 years, belonging to four families with three different mutations in *NLRP3*) was explored with pure tone audiograms, vestibular testing, and tinnitus questionnaires: 89% of them presented bilateral SNHL, which started at high frequencies and led to profound deafness in the most severe cases, and nearly half of the adults reported intermittent or permanent tinnitus [21]. Another study with additional cohorts of MWS patients reported a high percentage (67–92%) of hearing loss [22–24].

CINCA, also called neonatal-onset multisystem inflammatory disease (NOMID), was identified in 1987 [25] and represents the most severe phenotype of CAPS, with very early-onset skin rash, arthropathy and severe central nervous system symptoms, including chronic aseptic meningitis, which may lead to brain atrophy and severe intellectual disability [26]. Hearing loss is also a common symptom that occurs within the first years of life. Early anti-IL1B treatment is the standard therapy, reducing the risk of developing major complications.

CAPS is caused by single heterozygous germline or somatic gain-of-function mutations in the human *NLRP3* gene. The Infevers database (available at https://infevers.umai-montpellier.fr/, accessed June 17, 2025) currently lists 303 sequence variants of the *NLRP3* gene associated with autoinflammatory diseases, mostly substitutions affecting exon 4 (formerly named exon 3) [27], with pathogenic mutations typically located in the NACHT domain of the NLRP3 protein [28, 29]. Among them, 19 variants were specifically associated with CAPS (5 likely pathogenic, 3 variants of uncertain significance).

Genotype‒phenotype correlations are important for identifying predictive disease severity markers. Similarly, the Eurofever Registry analyzed 136 CAPS patients carrying *NLRP3* variants and concluded that skin rash, musculoskeletal involvement and fever were the most prevalent features, with neurological symptoms and hearing loss present in 40% and 42% of the patients, respectively [30]. Heterozygous germline mutations were found in 98% of the patients, and only 3 patients were mutation-negative despite complete *NLRP3* gene screening. Thirty-one different *NLRP3* gene mutations were detected, with 7 accounting for 78% of the patients, and 24 rare variants in 21% of the patients were significantly associated with early disease onset (< 6 mo), neurological complications or hearing loss. Specifically, rare variants linked to hearing loss included the T348M, V198M, E311K and A439V alleles. In addition, other variants, including R918Q, Y861C and Y861H and S595N, have been associated with atypical CAPS syndrome, with hearing loss as the primary presentation [5, 28, 29].

Although CAPS patients frequently suffer from sensorineural hearing loss, it remains unclear whether *NLRP3* mutation is the primary cause of cochlear autoinflammation, which may be the sole manifestation in some CAPS rare cases (DFNA34), or if systemic inflammation contributes to the development of progressive hearing loss. This fact may have an impact on treatment decisions. Notably, there is a window of opportunity to treat patients with anti-IL-1B, and younger patients are most likely to respond. Consequently, it is important to know the characteristics of CAPS for the early diagnosis of associated hearing loss, and mutation analysis of *NLRP3* will lead to a definite diagnosis [31].

In addition to CAPS, classical autoinflammatory diseases are characterized by apparently unprovoked inflammation without high-titer autoantibodies or antigen-specific T cells. These manifestations usually include neurological manifestations, such as meningitis, hearing loss, and other nonneurological manifestations. Among the genes involved in these diseases are those encoding *MEFV* (Mediterranean fever), *TNFR* (TNF receptor-associated periodic syndrome) or *MKV* (hyperimmunoglobulinemia syndrome). *NLRP3* mutations have also been identified. Thus, Salsano and coworkers demonstrated a novel *NLRP3* mutation (p.I288M) and a previously described MEFV mutation (p.R761H) in a patient with a chronic disease characterized by meningitis, osteomyelitis, leukoencephalopathy and progressive hearing loss, along with increased inflammatory markers. Patients respond to tocilizumab (an anti-IL-6 receptor monoclonal antibody) but not to anakinra (a recombinant IL-1R antagonist); therefore, IL-6 hypersecretion is the likely pathogenic mechanism [32].

### Nonsyndromic genetic hearing loss

*NLRP3* gene mutations are the cause of autosomal dominant autoinflammatory disorders, mostly CAPS, which include (syndromic) hearing loss. However, hearing loss has also been found to be the sole manifestation of these diseases, leading to a misdiagnosis of nonsyndromic deafness [33].

A variety of targeted NGS panels have been developed in recent years for genetic screening of nonsyndromic deafness, but they do not usually include typical syndromic deafness genes, such as *NLRP3*. Thus, Chen and colleagues conducted genetic screening via targeted next-generation sequencing (NGS) panels in a family with dominant inheritance initially diagnosed with nonsyndromic deafness. No pathogenic variants were found in any of the 72 known genes associated with nonsyndromic hearing loss. However, subsequent whole-exome sequencing identified a heterozygous p.E313K variant in the *NLRP3* gene. Follow-up clinical evaluation revealed that 6 out of 9 affected family members presented subtle inflammatory signs that had previously gone unnoticed [8].

Similarly, Nakanishi et al. identified a missense mutation, p.Arg918Gln, of the *NLRP3* gene associated with autosomal-dominant nonsyndromic SNHL in two unrelated families [5]. The affected subjects presented an atypical CAPS phenotype, with the sole symptom being a bilateral slowly progressive SNHL with an onset in the late 2nd to 4th decade of life that initially affects high frequencies, which can be improved or stabilized by anti-IL-1 therapy [31, 34].

In a recent study, 110 families with autosomal dominant hearing loss were tested with a custom panel of 237 hearing loss genes, and the *NLRP3* c.1872 C >G, p.Ser624Arg mutation was identified in one family [35]. ELISA and bioluminescence assays in peripheral blood mononuclear cells from these patients revealed that this novel gain-of-function mutation led to increased activity of caspase-1 and subsequent oversecretion of proinflammatory IL-1B [35]. Clinical reanalysis of the affected individuals, together with serological evidence of inflammation and pathological cochlear enhancement on magnetic resonance images, guided the diagnosis of atypical NLRP3 autoinflammatory disorder. In summary, genetic analysis in patients with nonsyndromic hearing loss should include genes causing these atypical forms to allow timely and effective treatment with IL-1 receptor antagonists.

### Nonhereditary congenital hearing loss

*Congenital cytomegalovirus infection.* It is the most common fetal viral infection and the leading nongenetic cause of SNHL in children, contributing to 25% of the cases under 4 years of age [36]. Cytomegalovirus infection induces a direct cytopathic effect in spiral ganglion neurons and a cochlear inflammatory response. A study with an experimental model of cytomegalovirus infection-associated hearing loss in newborn mice established that cytomegalovirus induced inflammasome-associated factors in spiral ganglion neurons and increased the content of reactive oxygen species [17]. More recently, cytomegalovirus has been shown to induce spiral ganglion neuron (SGN) death via both apoptosis and pyroptosis, with simultaneous activation of the p53/JNK and NLRP3/caspase-1 signaling pathways, respectively, due to the activity of the mixed lineage kinase family (MLK1/2/3), and the MLK inhibitor URMC-099 can prevent cytomegalovirus-induced SGN death and hearing loss [9].

*Bilirubin ototoxicity.* An increase in bilirubin levels in newborns can cause toxic effects on the auditory system, leading to hearing loss. Unconjugated bilirubin (UCB) can activate inflammatory mediators such as IL-18 and TNF, although the mechanism at the molecular and cellular levels remains unclear. Ex vivo organotypic cochlear cultures exposed to UCB presented demyelinated nerve fibers and a decreased size of spiral ganglion neurons, along with increased levels of NLRP3, cleaved caspase-1 and GSDMD. In addition, the application of pyroptosis inhibitors reduces the levels of the aforementioned proteins, ASC and IL-18, suggesting that the NLRP3 signaling pathway could be involved in UCB-induced ototoxicity [37].

### Drug ototoxicity

*Cisplatin-induced deafness.* Hearing loss is a serious secondary effect observed after antitumoral treatment with cisplatin, affecting 40–80% of adults and over 50% of children treated with this drug [38]. Cisplatin cytotoxicity is generally mediated through DNA crosslinking and reactive oxygen species production. The high susceptibility of the cochlea to cisplatin damage is due, in part, to long-term retention of cisplatin in the stria vascularis, where it induces an inflammatory response and marginal cell damage [39, 40].

A recent in vitro study confirmed that in response to cisplatin, marginal cells exhibit increased expression of NLRP3, caspase-1, IL-1B, and GSDMD, along with the formation of cell membrane pores. This situation was reversed by downregulation of NLRP3 by small interfering RNA, suggesting that NLRP3 inflammasome activation may mediate cisplatin-induced marginal cell inflammation and pyroptosis in the cochlear stria vascularis [11].

Additional mechanisms linking cisplatin ototoxicity to NLRP3 inflammasome activation have been described. First, cisplatin significantly decreased the levels of POU4F3, a transcription factor encoded by a well-known dominant nonsyndromic deafness pathogenic gene (DFNA15). Recently, Pou4f3 mutations were shown to promote cochlear hair cell pyroptosis by activating the NLRP3/caspase-3/GSDME pathway. Therefore, *Pou4f3* knockdown via shRNA can be combined with cisplatin treatment to induce pyroptosis in cochlear hair cells through the NLRP3/caspase-3/GSDME pathway [41]. Second, a retrospective cohort study with patients receiving cisplatin chemotherapy with or without concomitant antidepressive treatment revealed that the risk of ototoxicity was lower in the group treated with the selective serotonin reuptake inhibitors fluoxetine or fluvoxamine, which have been shown to inhibit the NLRP3 inflammasome [42]. In summary, the NLRP3 inflammasome plays a pivotal role in mediating cisplatin-induced ototoxicity through different mechanisms.

*Aminoglycoside ototoxicity.* Several antibiotics can induce hearing loss in children and adults, and the accumulation of oxygen radicals and inflammation in the inner ear are considered central pathological mechanisms. A recent study investigating whether the NLRP3 inflammasome is involved in aminoglycoside-related hearing loss revealed that mice treated with kanamycin plus furosemide presented increased levels of NLRP3 and increased levels of activated caspase-1, IL-1B, IL-18, and GSDMD-N and that oridonin treatment reversed this situation [16]. Furthermore, another study demonstrated that pharmacological inhibition of NLRP3 via MCC950, as well as genetic deletion of *NLRP3*, significantly protected against SGN degeneration in patients with aminoglycoside-induced hearing loss [43].

### Noise-induced hearing loss (NIHL)

Exposure to acute high-intensity noise can severely damage cochlear structures and induce the activation of DAMPs, which are recognized by innate immune receptors, triggering an inflammatory response [44]. Recent studies have confirmed increases in the levels of NLRP3, cleaved caspase-1, IL-1B, and IL-18 in the cochleae of minipigs and in mice exposed to 120 dB SPL noise, suggesting that the activation of the NLRP3 inflammasome constitutes a central pathogenic mechanism in NIHL [15, 45]. Moreover, the use of anakinra or oridonin has been proven to be effective in protecting mice from NIHL by facilitating inflammasome complex assembly [45, 46].

Chronic exposure to moderate levels of noise also affects the inner ear and reorganizes central auditory pathways, although the role of NLRP3 remains to be elucidated. In a study from Feng and collaborators, C57BL/6J mice were exposed to long-term 70 dB SPL white noise, aggravating the concomitant age-related hearing impairment typical of this strain [47]. They reported that cochlear ribbon synapses were the primary site of inner ear injury caused by chronic noise exposure. These authors confirmed by western blotting the presence of a significant increase in the levels of NLRP3, caspase-1 and IL-1B in P3 mouse cochlear explants exposed to NMDA and kainate to mimic noise-induced excitotoxic damage. These results indicate that NLRP3 inflammasome is an important mechanisms underlying auditory nerve fiber damage after noise [47].

### Age-related hearing loss (ARHL)

ARHL, or presbyacusis, is a progressive loss of hearing sensitivity predominantly associated with hair cell and SGN degeneration in the inner ear. Oxidative stress and a chronic low-level inflammatory response are frequently found in aging cochleae. Although inflammasomes are likely responsible for the accumulation of reactive species in immune cells, whether they are involved in the development of ARHL is still unknown. A study in mice demonstrated via RT‒qPCR, western blotting and ELISA that the levels of activated NLRP3, caspase 1, IL-1B and IL-18 were significantly greater in the inner ears of aged mice than in those of young mice [48].

### Meniere’s disease

Meniere’s disease is an inner ear disorder characterized by severe vertigo episodes and hearing loss. The causes and precise pathological mechanisms remain undefined, although alterations in immune responses have been proposed. Recently, downregulation of serum/glucocorticoid-inducible kinase 1 (SGK1) was shown to be associated with activation of the NLRP3 inflammasome in vestibular resident macrophage-like cells from Meniere’s disease patients [49]. Moreover, *Sgk*^*−/−*^ mice that received LPS presented severe audiovestibular symptoms, increased inflammasome activation and endolymphatic hydrops, which were ameliorated by blocking NLRP3. Pharmacological inhibition of SGK 1 also increases disease severity in vivo. SGK1 phosphorylates the NLRP3 PYD domain, which acts as a physiological inhibitor of NLRP3 inflammasome activation to maintain inner ear immune homeostasis. SGK1 depletion enhances the NLRP3 inflammasome and IL-1B production, potentially leading to damage to inner ear hair cells and the vestibular nerve. Thus, SGK1 inhibition could offer an alternative to current treatments based on corticosteroid administration [50].

### Vestibular Schwannoma

Vestibular schwannomas arise from neoplastic Schwann cells of the vestibular nerve and constitute the fourth most common type of intracranial tumor, often causing SNHL and tinnitus [51]. There was no correlation between tumor size and the grade of hearing loss, suggesting that vestibular schwannoma-associated SNHL is due not only to mechanical compression of the auditory nerve but also to differences in the intrinsic biology of these tumors. Previous research has reported an abnormal upregulation of inflammatory pathways in these tumors and a correlation between poor hearing and a robust inflammatory response in vestibular schwannoma patients. A meta-analysis of a large vestibular schwannoma microarray dataset by Sagers and collaborators identified the NLRP3 inflammasome as a candidate, which was further validated in human vestibular schwannoma tissue via RT‒qPCR and immunohistochemistry [52]. In addition, the authors reported an association between the overexpression of NLRP3 inflammasome components in vestibular schwannoma and a high degree of hearing loss. Therefore, the inhibition of the NLRP3 inflammasome in vestibular schwannoma could contribute to preserving hearing.

## Auditory function in Nlrp3 mutant mice

The pathogenesis of hearing loss due to inflammasome activation remains incompletely understood. Therefore, animal models are still necessary to obtain key information. According to the Mouse Genome Informatics database, 47 *Nlrp*3 mutant alleles were generated, 18 by classical gene targeting, 18 by endonucleases and 13 induced chemically (URL: http://www.informatics.jax.org, accessed June, 2025). Most of them are knockouts (null or conditional), but others incorporate humanized sequences or include mutations found in the *NLRP3* gene in patients suffering from autoinflammatory disorders (Table 1).

**Table 1:** *Nlrp3* mutant alleles

Mutant allele	Mutation details	Abnormal phenotype	Human disease	Reference
*Nlrp3* ^*tm1Hhf*^ NLR family, pyrin domain containing 3; targeted mutation 1, Hal M Hoffman *(Nlrp3* ^*A350VneoR*^*)*	Modeling the A352V human mutation linked to Muckle-Wells syndrome (MWS), A floxed neomycin resistance cassette was inserted in reverse orientation into intron 2, upstream of the mutated exon. Upon Cre-mediated recombination, the cassette is excised, allowing expression of the mutant allele.	digestive/alimentary, growth/size/body, hematopoietic, homeostasis, immune, integument, mortality/aging, renal/urinary	MWS	[53]
*Nlrp3* ^tm2Hhf^ NLR family, pyrin domain containing 3; targeted mutation 2, Hal M Hoffman *(Nlrp3* ^*L351PneoR*^*)*	Modeling the human L353P mutation associated with familial cold autoinflammatory syndrome (FCAS). A floxed neomycin resistance cassette was inserted in reverse orientation into intron 2, upstream of the mutant exon. Upon Cre recombinase expression, the cassette is excised, allowing expression of the mutant allele.	hematopoietic, immune, mortality/aging	FCAS	[53]
*Nlrp3* ^*tm1Smoc*^ NLR family, pyrin domain containing 3; targeted mutation 1, Shanghai Model Organisms Center *(Nlrp3* ^*tm(LSL−A350V)Smoc*^ *)*	This allele carries a A350V conditional mutation of the gene			Shanghai Model Organisms Center,
*Nlrp3* ^*tm1Wstr*^ NLR family, pyrin domain containing 3; targeted mutation 1, Warren Strober *(Nlrp3* ^*R258W*^ *)*	To model the human R260W *NLRP3* mutation linked to Muckle-Wells syndrome (MWS), mice were engineered with the equivalent R258W mutation. A neomycin resistance cassette flanked by FRT sites and a pair of loxP sites were inserted around exon 3. When Cre recombinase is expressed, exon 3 is deleted, resulting in a frameshift mutation that creates a null allele.	growth/size/body, hematopoietic, immune, integument, liver/biliary, mortality/aging, reproductive	MWS	[54]
*Nlrp*3^tm3.1Hhf^ NLR family, pyrin domain containing 3; targeted mutation 3.1, Hal M Hoffman (D301N NLRP3, NOMID)	To model the D301N mutation associated with CAPS, researchers introduced a point mutation into exon 3 of the mouse *Nlrp3* gene, corresponding to human residue 303. A neomycin resistance cassette flanked by loxP sites was inserted in reverse orientation into intron 2. After Cre recombinase excised the cassette, the mutant allele was expressed.	cellular, growth/size/body, hematopoietic, homeostasis, immune, limbs/digits/tail, mortality/aging, nervous system, skeleton	CINCA	[55]
*Nlrp3* ^tm3.1(NLRP3*)Bhk^ NLR family, pyrin domain containing 3; targeted mutation 3.1, Beverly H Koller D305N	Humanized mouse model generated by replacing the mouse *Nlrp3* gene with a human variant carrying the CAPS-associated p.Asp303Asn mutation. A vector containing the human gene and a mutated loxP site was introduced into Nlrp3 < sup > tm1Bhk</sup >embryonic stem cells, where the original locus had been replaced by a neomycin cassette flanked by FRT sites. After Flp recombination removed the cassette, Cre recombinase enabled targeted insertion of the mutant human gene at the endogenous locus, restoring gene expression.		CAPS	[56]
*Nlrp3*_c.2750 G >A EM:15,753	Mice generated by CRISPRCas9 edition, carrying a specific point mutation in the *NLRP3* gene, where at DNA position 2750 a guanine (G) is replaced by an adenine (A) resulting in a missense variant, An equivalent missense mutation, p.Arg918Gln (c.2753G >A) causes autosomal-dominant sensorineural hearing loss DFNA34 in two unrelated families single-nucleotide variant has been identified in patients	Ongoing	DFNA34	[5]

Table 1. Nonexhaustive list of *Nlrp3* mouse mutant alleles generated, including pathogenic human *NLRP3* variants identified in CAPS patients. Source: Mouse Genome Informatics Database and INFRAFRONTIER/EMMA repository. Accessed June 2025.

The first knockout avatar mice expressing *Nlrp3* gain-of-function mutations identified in MWS (A352V, R258W) and FCAS (L353P) patients presented with systemic inflammation, poor growth, and increased mortality rates before weaning; therefore, these mice did not survive to an age when hearing evaluation was possible [53, 54]. Similarly, a mouse expressing the D301N *NLRP3* mutation (the ortholog of D303N in human *NLRP3*, which causes CINCA) was subsequently generated. These mice exhibit neutrophilia and high levels of serum inflammatory mediators, along with abnormalities in postnatal skeletal growth and bone remodeling, analogous to those observed in CINCA patients. They also exhibit growth retardation and lower body weight and usually die by 2–3 weeks of age [55].

In 2016, Snouwaert and collaborators generated a mouse mutant carrying a D305N SNP in the *Nlrp3* gene (autoinflammatory disease-associated *NLRP3* D303N SNP). These mice present a normal appearance at birth and early growth, surviving beyond weaning. Later, they develop systemic inflammatory symptoms, including splenomegaly, with a marked increase in myeloid cells in the spleen, blepharitis, and meningeal inflammation. Arthritic changes became apparent within the first 3 months of life and progressed as the mice aged, along with osteoporosis and kyphosis. Nevertheless, no evidence of hearing loss was detected [56].

In 2022, Kim and collaborators bred *Nlrp3*^*D301NneoR*^ mice [55] (available at The Jackson Laboratory, Jax #017971) with a *Gfi1-Cre* knock-in mouse line for the activation of this conditional mutant *NLRP3* in the cochlea and hematopoietic cells, bypassing preweaning mortality [57]. This novel mouse model exhibited severe to profound hearing loss at postnatal day 20 and cochlear inflammation detected by MRI, along with the overexpression of mutant NLRP3 in the spiral prominence, inner and outer sulcus regions, organ of Corti, and SGN. The cochleae of these mice at P12 presented a disorganized organ of Corti and a collapsed Nuel’s space. They also show varying degrees of inflammation with lymphocytic infiltration in the brain, kidney, and liver [57]. A similar *Nlrp3*^*D301NneoR/Flox*^ *Cx3cr1*^*CreER7+*^ mouse model with conditional expression of the *Nlrp3* D301N mutation in CX3CR1-positive cells (macrophage and microglia) was generated by Ma and collaborators. Compared with control mice, these mice presented more severe cochlear inflammation, inflammasome activation and hearing loss after LPS injection. These symptoms are reduced by the administration of the NLRP3 inhibitor MCC950 [58]. These mouse lines model human autoinflammatory hearing loss and could be valuable tools for elucidating the underlying pathogenic mechanism of inflammasome activation-mediated hearing loss. Similarly, our group recently generated a CRISPR-Cas9 mouse mutant that reproduces the missense mutation c.2753G >A (p.Arg918Gln) of *NLRP3*, which causes the nonsyndromic autosomal-dominant sensorineural hearing loss DFN34 [5].

## NLRP3 inflammasome inhibitors as drugs for hearing loss treatment

FDA and/or EMA-approved inhibitors of IL-1B signaling, such as anakinra, canakinumab or rilonacept, are the first-line therapeutic options available for CAPS and other anti-inflammatory diseases [18]; therefore, they can benefit patients with associated hearing loss.

Anakinra (Kineret^®^) is a modified version of the human IL-1R antagonist protein that competitively inhibits the binding of IL-1 A and IL-1B to the IL-1R1 receptor, thereby reducing inflammation and tissue damage. It is produced in *E. coli* via recombinant DNA technology and is commonly used to treat CAPS via subcutaneous injection.

A recent study revealed its potential to treat human hearing loss in select cases of NLRP3-related autoinflammatory disorders. Thus, a significant improvement in hearing after anakinra therapy was observed in 17 families diagnosed with either CAPS or DFNA34, although the *NLRP3* genotype, hearing status at diagnosis and cochlear radiological findings were prognostic factors for the final hearing status after treatment [59]. For patients suffering severe deafness not responsive to anakinra, cochlear implantation could be the last option for hearing rehabilitation. Although not generally indicated for patients with autoinflammatory diseases, recent successful outcomes have been reported for cochlear implants in CINCA, MWS and DFNA34 patients [60].

Anakinra has been shown to partially alleviate the degree of hearing impairment in a NIHL mouse model, suggesting that inhibiting NLRP3 and downstream signaling pathways may constitute a new strategy for the clinical treatment of this condition.

Canakinumab (Ilaris^®^) is a fully human monoclonal IgG1 anti-IL-1B antibody that provides selective and prolonged IL-1B blockade and a rapid (within hours), complete and sustained response in most CAPS patients, without any consistent pattern of side effects. Long-term follow-up trials have demonstrated the sustained efficacy, safety and tolerability of canakinumab [61]; therefore, it was approved by the FDA for FCAS and MWS and by the EMA for the treatment of all three CAPS phenotypes. With respect to SNHL, the efficacy of canakinumab is variable, as reported in an MWS family, where only the younger member showed auditory improvement, highlighting the importance of early intervention [62].

Gevokizumab is an experimental (not yet approved) monoclonal antibody that selectively neutralizes IL-1B and has been explored as a potential treatment for autoimmune inner ear diseases resistant to corticosteroids in a phase 2 clinical study (NCT01950312), with no updated results [63]. Similarly, rilonacept (Arcalyst^®^) is a soluble IL-1R that blocks IL-1 A and IL-1B signaling and has not yet been widely established for SNHL treatment. An early phase 1 proof-of-concept clinical trial (NCT02828033) was initiated in 2017 to explore rilonacept in patients with autoimmune SNHL, but no results have been reported to date [63].

The majority of current knowledge on the role of NLRP3 in hearing loss stems from animal studies; therefore, several therapeutic strategies specifically targeting NLRP3 itself have been tested preclinically and are progressing toward clinical translation.

Piceatannol [(E)−4-(3,5-dihydroxystyryl) benzene-1,2-diol] is a natural analog of resveratrol that has been shown to have immunomodulatory and anti-inflammatory effects, among other activities. A recent study investigated its effect in ARHL models both in vitro and in vivo. Thus, HEI-OC-1 cells exposed to LPS to simulate the aging inflammatory environment presented increased expression of NLRP3, caspase-11 and GSDMD, whereas treatment with PCT reduced inflammation-associated protein expression and pyroptosis, improving cell survival. In vivo experiments revealed that PCT protects mice from ARHL and reduces inner hair cell and spiral ganglion neuron loss. These results suggest a protective role for PCT against ARHL, possibly through the caspase-11-GSDMD pathway [64].

Oridonin is a traditional medicinal product with anti-inflammatory properties that has been recently reported to protect mice from NIHL. Li et al. confirmed that oridonin acts by blocking the interaction between NLRP3 and NEK7 (NLRP3 never in mitosis gene A-related kinase 7) and therefore inflammasome complex assembly, which inhibits the downstream inflammasome. Another factor regulated by oridonin that could explain the otoprotective effect after noise exposure is IL-1R type 2 (IL-1R2). AAV-mediated overexpression of IL-1R2 in the inner ear significantly reduces NIHL and ribbon synapse lesions by blocking cytokine storms; similarly, oridonin induces IL-1R2 expression in spiral ganglion neurons and hair cells.

Oridonin has also shown efficacy in treating aminoglycoside ototoxicity, reducing kanamycin-related hearing loss by inhibiting NLRP3 inflammasome activation and caspase-1/GSDMD-related hair cell pyroptosis. These findings demonstrate that pyroptosis, as well as apoptosis, may be involved in kanamycin-induced hearing loss and that the NLRP3 inflammasome could be a new target for treating aminoglycoside ototoxicity [16].

MCC950 [{N-[(1,2,3,5,6,7-hexahydro-s-indacen-4-yl) carbamoyl]−4-(2-hydroxypropan-2-yl)furan-2-sulfonamide}] is a chemically characterized NLRP3 inhibitor that has been shown to be effective in different inflammatory models, such as CAPS and gout.

With respect to hearing loss, Ma and colleagues reported that MCC950 significantly alleviated systemic LPS-induced hearing loss and the inflammatory phenotype in mutant mice expressing the pathological *NLRP3* variant D301N in cochlea-resident CX3CR1 macrophages.

Dapansutrilo (OLT1177, [3-(methylsulfonyl)propanenitrile]) is a selective NLRP3 inflammasome inhibitor that is currently under investigation for various inflammatory conditions, including gout and cardiovascular disease. It is gaining attention for its potential to modulate IL-1B-driven inflammation and is currently being evaluated in phase I and II clinical trials [63].

Finally, tranylcypromine, a monoamine oxidase inhibitor traditionally used as an antidepressant, has been recently proposed to have otoprotective effects in a mouse model of NIHL via different mechanisms, including blockade of NLRP3 inflammasome signaling [65]. Table 2 summarizes key therapeutic interventions for NLRP3-related SNHL, their mechanisms, indications, and clinical development stages.

**Table 2:** Treatments for NLRP3 autoinflammatory disorders

Name	Mechanism of Action	Indication	Current Status
Anakinra	IL-1R antagonist that blocks IL-1B signaling, reducing downstream inflammation	Autoinflammatory CAPS-related sensorineural hearing loss; mitigates Noise-induced hearing loss in animals	Approved (FDA/EMA) therapy for CAPS
Canakinumab	Monoclonal antibody that neutralizes IL-1B to prevent inflammasome-mediated inflammation	CAPS-related sensorineural hearing loss (e.g., MWS, NOMID)	Approved (FDA/EMA)
Rilonacept	Decoy receptor fusion protein for IL-1B and IL-1 A	NLRP3-associated autoinflammatory hearing loss	Approved (FDA)
Cochlear Implant	Bypasses damaged cochlear cells with direct electrical stimulation of the auditory nerve	Severe sensorineural hearing loss in CAPS/DFNA34 unresponsive to drugs	Standard clinical medical device for SNHL
Piceatannol	Polyphenol (resveratrol analog) that reduces NLRP3, caspase-11, and GSDMD; inhibits pyroptosis	Age-related hearing loss	Preclinical
Oridonin	Blocks NLRP3–NEK7 interaction and IL-1B release	Noise-induced and aminoglycoside-induced hearing loss	Preclinical
MCC950	Selective small-molecule NLRP3 inhibitor	Inflammation-driven sensorineural hearing loss (e.g., CAPS models)	Preclinical
Tranylcypromine	LSD1 inhibitor that upregulates SESN2, activates autophagy, and suppresses NLRP3	Noise-induced hearing loss	Preclinical (drug repurposing)
Dapansutrilo (OLT1177)	Oral selective NLRP3 inhibitor; demonstrated reduction of IL-1B/IL-18 in preclinical models; well-tolerated in Phase 1 trials	Inflammation-related sensorineural hearing loss	Early clinical development (Phase 1 completed; Phase 2 ongoing in gout and heart failure)

Table 2. List of therapeutic approaches used to treat NLRP3 autoinflammatory disorders. Sources: ClinicalTrials.gov and Euclinicaltrials.eu, accessed June 2025.

## Conclusions

The growing body of evidence reviewed here firmly establishes the NLRP3 inflammasome as a pivotal player in the pathogenesis of sensorineural hearing loss across multiple etiologies. From inherited autoinflammatory syndromes to noise exposure, aging and ototoxic insults, NLRP3 activation is consistently correlated with increased cochlear inflammation, pyroptotic cell death and hearing loss. Genetic studies further implicate *NLRP3* variants in both syndromic and nonsyndromic progressive hearing loss. These studies also revealed a spectrum of disease phenotypes responsive to IL-1B inhibition. While IL-1R1 receptor antagonists and monoclonal antibodies remain the basis of clinical therapy for CAPS-related deafness, the development of direct NLRP3 inhibitors, some of which are already in early clinical stages, offers a promising horizon for broader applications. Advances in animal modeling, drug screening and molecular imaging are expected to deepen our understanding of the role of NLRP3 in hearing receptors.

Future research should prioritize translational efforts to validate NLRP3-targeting compounds in human trials, explore biomarkers for early diagnosis, and investigate combination therapies that integrate anti-inflammatory, antioxidant and regenerative strategies. Ultimately, targeting the NLRP3 inflammasome may redefine therapeutic paradigms for preventing or halting irreversible hearing loss.

## Data Availability

No datasets were generated or analysed during the current study.

## Abbreviations

ARHL: Age-related hearing loss
ASC: Apoptosis-associated speck-like protein containing a CARD
CAPS: Cryopyrin-associated Periodic Syndromes
CINCA: Chronic infantile neurological, cutaneous and articular
DAMP: Damage-associated molecular pattern
DFNA: Autosomal dominant nonsyndromic deafness
FCAS: Familial cold autoinflammatory syndrome
GSDM: Gasdermin
HEI-OC1: House Ear Institute Organ of Corti-1 cell line
IL-1B: Interleukin 1 beta
IL-1R: Interleukin 1 beta receptor
IL-18: Interleukin 18
LPS: Lipopolysaccharide
MWS: Muckle–Wells syndrome
NLRP3: NOD-, LRR- and pyrin domain-containing protein 3
PAMPs: Pathogen-associated molecular patterns
SGN: Spiral ganglion neuron
SNHL: Sensorineural Hearing Loss
